# Supplementary material for: Finding a Secure Place in the Home during the First COVID-19 Lockdown: A Pattern-Oriented Analysis
Source: Behav Sci (Basel). 2022 Dec 22;13(1):9. doi: 10.3390/bs13010009 (PMC9854664; doi:10.3390/bs13010009)
Supplement: Supplementary file 1 [file behavsci-13-00009-s001.zip › behavsci-1965022-supplementary.pdf]

Table S1: Fit indices of the LPA models

	**Model1**				**Model2**				**Model3**				**Model6**			
	**AIC**	**BIC**	**entropy**	**BLRT / p**	**AIC**	**BIC**	**entropy**	**BLRT / p**	**AIC**	**BIC**	**entropy**	**BLRT / p**	**AIC**	**BIC**	**entropy**	**BLRT / p**
1	8605	8642	1.00		8605	8642	1.00		8573	8638	1.00		8573	8638	1.00	
2	8425	8485	0.85	128.8 (<.01)	7960	8039	0.68	663.2 (<.01)	8204	8292	0.96	119.9 (<.01)	7911	8046	0.74	240.6 (<.01)
3	8054	8138	0.91	95.0 (<.01)	7848	7968	0.68	130.1 (<.01)	8178	8289	0.73	58.4 (<.01)	7600	7803	0.79	63.7 (<.01)
4	8157	8264	0.67	0.0 (1.00)	**7594**	**7756**	0.68	272.2 (<.01)	8147	8281	0.67	0 (<.01)	7495	7768	0.74	98 (<.01)
5	8024	8153	0.68	9.5 (<.01)	7620	7824	0.70	**-8.7 (1.000)**	8022	8179	0.67	-0.1 (<.01)	7455	7798	0.73	46.3 (<.01)
6	8034	8186	0.57	44.0 (0.87)	**7503**	**7749**	0.74	135.0 (<.01)	8032	8212	0.57	47.2 (0.85)	7405	7817	0.74	-41.4 (<.01)
7	8008	8184	0.58	26.0 (<.01)	7501	7788	0.73	20.5 (0.18)	8042	8245	0.51	51.1 (0.87)	7403	7884	0.77	97.5 (0.20)
8	8011	8210	0.57	42.2 (0.29)	*	*	*	*	8048	8275	0.51	38.8 (0.60)	7371	7922	0.78	50.7 (<.01)

Note: bold values represent local minima for AIC and BIC and the first non-significant BLRT value

Model 1: equal variances and zero covariances; Model 2: varying variances and zero covariances; Model 3: equal variances and covariances; Model 4: varying variances and covariances

* Model 2 with eight latent profiles did not converge
